# Prolactin Drives Iron Release from Macrophages and Uptake in Mammary Cancer Cells through CD44

**DOI:** 10.3390/ijms25168941

**Published:** 2024-08-16

**Authors:** Reagan Farrell, Nicholas Pascuzzi, Yi-Ling Chen, Mary Kim, Miguel Torres, Lauren Gollahon, Kuan-Hui Ethan Chen

**Affiliations:** 1 Department of Biological Sciences, Texas Tech University, Lubbock, TX 79409, USA; reagan.farrell@ttu.edu (R.F.); npascuzz@ttu.edu (N.P.); tor46402@ttu.edu (M.T.); lauren.gollahon@ttu.edu (L.G.); 2 Department of Electronic Engineering, National Kaohsiung University of Science and Technology, Kaohsiung 80778, Taiwan

**Keywords:** prolactin, CD44 upregulation, iron transfer, macrophages, mammary cancer cells

## Abstract

Iron is an essential element for human health. In humans, dysregulated iron homeostasis can result in a variety of disorders and the development of cancers. Enhanced uptake, redistribution, and retention of iron in cancer cells have been suggested as an “iron addiction” pattern in cancer cells. This increased iron in cancer cells positively correlates with rapid tumor growth and the epithelial-to-mesenchymal transition, which forms the basis for tumor metastasis. However, the source of iron and the mechanisms cancer cells adopt to actively acquire iron is not well understood. In the present study, we report, for the first time, that the peptide hormone, prolactin, exhibits a novel function in regulating iron distribution, on top of its well-known pro-lactating role. When stimulated by prolactin, breast cancer cells increase CD44, a surface receptor mediating the endocytosis of hyaluronate-bound iron, resulting in the accumulation of iron in cancer cells. In contrast, macrophages, when treated by prolactin, express more ferroportin, the only iron exporter in cells, giving rise to net iron output. Interestingly, when co-culturing macrophages with pre-stained labile iron pools and cancer cells without any iron staining, in an iron free condition, we demonstrate direct iron flow from macrophages to cancer cells. As macrophages are one of the major iron-storage cells and it is known that macrophages infiltrate tumors and facilitate their progression, our work therefore presents a novel regulatory role of prolactin to drive iron flow, which provides new information on fine-tuning immune responses in tumor microenvironment and could potentially benefit the development of novel therapeutics.

## 1. Introduction

Iron is an essential element for human health. In humans, about two-thirds of the body iron is found in circulating erythrocytes as part of hemoglobin [1]. The remaining one-third of the body iron is contained/carried by transferrin or ferritin as a readily mobilizable iron storage and/or incorporated into proteins for a variety of cellular functions [1]. One major function of iron in cells is its direct involvement in the formation of several iron-sulfur containing complexes for cellular respiration [2,3]. Iron is also a critical component of many heme-containing enzymes involved in oxidative metabolism [4,5,6]. Dysregulated iron homeostasis is associated with multiple human diseases. Iron deficiency leads to anemia [7], while iron excess can result in a variety of neurological disorders as diverse as multiple sclerosis, stroke, and Parkinson’s disease [8,9]. Iron excess also promotes infections and the development of cancers [10,11,12,13,14]. 

Recently, enhanced iron uptake, redistribution, and retention in cancer cells has been observed, suggesting an “iron addiction” behavior in cancer cells [15,16,17,18,19]. Sufficient iron in cancers promotes the catalytic function of ribonucleotide reductase, a key enzyme for DNA synthesis during cell replication [20]. Iron deficiency in cancers causes cell cycle arrest through the induction of the cell cycle inhibitory regulators p15, p21, and p27 [21]. In addition, iron depletion from multiple mouse models of lung, ovarian, mammary, and prostate tumors decrease tumor metastasis and increase tumor cell survival [22,23,24,25]. Evidence from a variety of cancer types even suggests that iron addiction could be more pronounced in cancer stem cells [18,26,27,28,29]. However, it has also been reported that iron overload could be toxic to cells, including cancer cells, and cause ferroptosis [18,30]. Thus, a better understanding of iron metabolism in cancer cells could provide clues for future therapeutic designs.

Prolactin is a lactogenic hormone generated and released by the anterior pituitary [31]. As its name implies, the primary function is to promote lactation. While the synthesis of prolactin by pituitary lactotrophs during pregnancy and subsequent lactation is well studied [32,33], its expression independent of pregnancy and lactation has received far less attention. Outside of the pituitary, a small amount of prolactin can be produced by non-lactotrophs, including mammary epithelia and a variety of cancer cells as an autocrine survival factor [34,35,36]. The dark side of prolactin in promoting cancer progression has been well documented in multiple cancers [37,38,39,40,41,42]. Prolactin promotes proliferation and migration of cancer cells, enhances tumor stemness, and drives tumor metastasis [37,43,44,45]. In addition, prolactin, together with leptin, osteopontin, and insulin-like growth factor 2, provides high sensitivity and specificity for early-stage cancer prediction [46]. 

Interestingly, iron deficiency is associated with increased serum prolactin and/or even hyperprolactinemia [47,48]. This elevation is, in part, caused by dysfunction of neurons that produce dopamine, the primary tonic inhibitor of prolactin release from the anterior pituitary [49,50]. As prolactin is a stress hormone and the secretion of prolactin often acts to maintain homeostasis [51,52], it is important to elucidate whether such elevation of prolactin in response to iron deficiency provides any feedback or tolerance to cells. Additionally, the role of prolactin in iron uptake, redistribution, and retention at the cellular level is also unknown.

In the present study, we show distinct responses in iron transport due to prolactin treatment in two types of cells: mammary cancer cells and immune macrophages. In the mammary cancer cells, prolactin stimulation leads to labile iron pool accumulation. This elevation of the labile iron pool is mediated by the upregulation of the surface receptor CD44, which is known to initiate endocytosis of iron bound by hyaluronate [27]. In contrast, in the macrophages, prolactin causes iron export through the induction of ferroportin, the main iron exporter. Interestingly, when macrophages and mammary cancer cells were co-cultured, we observed a direct labile iron pool transfer from macrophages to the cancer cells with prolactin treatment. Consequently, our results demonstrate a novel role of prolactin in the regulation of iron transport and provide first-hand evidence for the direct transfer of iron from macrophages to cancer cells. These results advance our understanding of the tumor-immuno-microenvironment and may contribute to the development of novel treatment targets that will complement current therapeutics.

## 2. Results

### 2.1. Prolactin Drives Iron Accumulation in Mammary Cancer Cells

**Figure 1:**
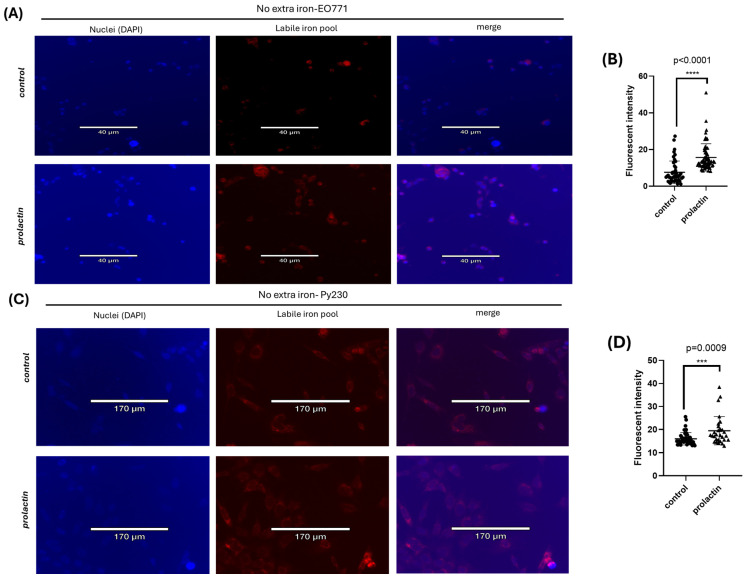
Regulation of intracellular labile iron pool by prolactin in mouse breast cancer cells under normal culture condition. Effect of prolactin treatment on the intracellular labile iron pool in EO771 cells (**A**) and Py230 cells (**C**). Blue and red fluorescence stains of the nucleus and labile iron pool, respectively. Quantification of the intracellular labile iron fluorescent intensity by prolactin treatment in EO771 cells (**B**) and Py230 cells (**D**). Results are calculated as the average labile iron fluorescence of all individual cell from three independent trials (*n* = 3). Data represent mean ± SD. (***, *p* < 0.001; ****, *p* < 0.0001).

Elevated serum prolactin is associated with increased risks of cancers [53,54,55]. To mimic prolactin elevation, mouse mammary cancer cells, EO771, were treated with 100 ng/mL prolactin for 2 days. The impact of prolactin on iron acquisition was evaluated by FerroOrange staining to detect the presence and amount of the labile iron pool. Under normal culture conditions (no extra iron supplement), the major iron source was the fetal bovine serum (~2.4 μg/mL) in the culture medium. Prolactin treatment led to a significant increase in the intracellular labile iron pool (Figure 1A, and quantification in Figure 1B). To further confirm the role of prolactin in the regulation of iron transport in mammary cancer cells, performance of the same iron uptake experiments was used with a different mouse triple negative mammary cancer cell line, Py230. Prolactin stimulation again led to increased accumulation of labile iron pool (Figure 1C, and quantification in Figure 1D). The numerical number of fluorescent quantifications for EO771 cells is also presented in Table 1. 

Dietary iron may exist in the oxidized ferric (Fe^3+^) state or the reduced ferrous (Fe^2+^) state, depending on the environmental pH. To investigate if prolactin treatment impacted iron uptake with extra iron supplementation, and with different re/dox states of iron, EO771 cells were treated with prolactin along with extra ferric or ferrous ions (0.1 mg/mL). Results showed a potentially biphasic role of prolactin in the regulation of iron uptake. Under normal iron concentration (no extra iron supplementation), prolactin positively impacted the iron uptake in mammary cancer cells while prolactin treatment might protect EO771 cells from iron overload in the presence of excess iron regardless of the redox state of iron (Supplementary Figure S1A with excess ferrous iron and Supplementary Figure S1B with excess ferric iron). The intracellular iron staining was overwhelmed when extra ferric iron was provided. Although EO771 cells showed a preference for ferric iron uptake based on the intracellular iron staining intensity, the addition of extra ferrous or ferric iron had no significant effect on tumor cell proliferation over a 4-day period (Supplementary Figure S1E,F). In Py230 cells, prolactin again decreased iron uptake in the presence of extra ferric iron (Supplementary Figure S2B) but there was a slight increase in the level of labile iron in prolactin-stimulated Py230 cells with extra ferrous iron (Supplementary Figure S2A), suggesting differential sensitivity to environmental iron concentrations between different tumor cell lines.

Taken all together, these results demonstrated that mammary cancer cells acquired iron from the environment and the peptide hormone prolactin positively regulated iron uptake into cancer cells under normal circumstances (no extra iron presence). When excess iron was present, prolactin might exhibit a protective role when exceeding iron concentration that cells can tolerate.

### 2.2. Upregulation of CD44 by Prolactin Stimulation May Contribute to Iron Accumulation in Mammary Cancer Cells

**Figure 2:**
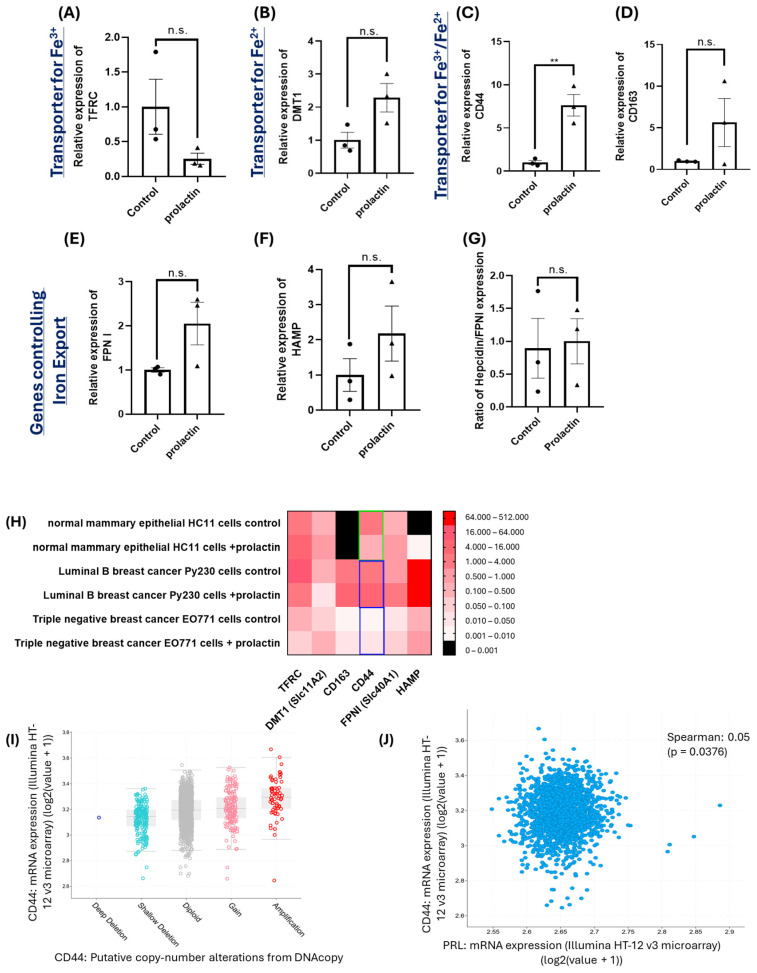
Effects of prolactin treatment on gene expression involved in iron transport in E0771 cells. Changes in expression levels in the Fe^3+^ transporter TFRC (**A**), Fe^2+^ transporter DMT1 (**B**), Fe^3+^ and Fe^2+^ transporters including the hyaluronate receptor CD44 (**C**) and scavenger receptor CD163 (**D**), iron exporter FPN I (**E**), the negative regulator hepcidin for iron exporter (**F**), and the ratio of hepcidin to FPN I (**G**). Data represent mean ± SEM from three biological replicates (*n* = 3) (**, *p* < 0.01; n.s. = not significant). Comparison of iron transport-related gene expression between normal and breast cancer cell lines (**H**). The green and blue rectangles indicate downregulation and upregulation of the CD44 gene, respectively. Analysis of 2326 human patients with invasive breast carcinoma revealed significant types of CD44 mutations (x-axis) and its overexpression (y-axis) (**I**). Among these patients, a positive correlation (Spearman coefficient = 0.05, *p* = 0.04) was found between tumoral expression of CD44 (y-axis) and prolactin (x-axis) (**J**).

Cells acquire ferric (Fe^3+^) and ferrous (Fe^2+^) ions through different pathways. Free or transferrin-bound ferric ions are transported into cells via transferrin receptor (TFRC) [56,57]. Ferric ions can also be reduced to ferrous ions by STEAP, a ferrireductase, and transported into cells via divalent metal-ion transporter 1 (DMT1) [58]. Iron (either reduced Fe^2+^ or oxidized Fe^3+^) that is present in hemoglobin/haptoglobin or bound by hyaluronate is taken into cells via scavenger receptors CD163 and CD44, respectively [27,59]. Additionally, iron export is accomplished by ferroportin (FPN I), which is the only exporter and is negatively regulated by the molecule hepcidin (HAMP) [60,61]. Based on these previous findings, the next determination was whether the accumulated labile iron pool produced by prolactin stimulation was an outcome of increased uptake of ferric, ferrous, or both ions and/or the decreased regulation of iron export through FPN I and hepcidin. To that end, EO771 cells were treated by prolactin (100 ng/mL) for 2 days and expression of genes involved in iron transport was analyzed using real-time qPCR. While there was a trend of downregulation of the Fe^3+^ transporter TFRC, by prolactin stimulation, this was not statistically significant (Figure 2A). There was also non-significant increase of gene expression by prolactin in DMT1, which transported extracellular ferrous ions into cells or from the endosome into the cytoplasm (Figure 2B), and CD163 which mediated the transport of iron contained in hemoglobins/haptoglobins (Figure 2D). Interestingly, prolactin treatment increased CD44, which is known to initiate the endocytosis of hyaluronate bound iron (Figure 2C). There was no difference in the FPN I (Figure 2E) and hepcidin (Figure 2F) expression by prolactin stimulation. The ratio of hepcidin/FPN I expression was not different between control and prolactin treatment in EO771 cells (Figure 2G), suggesting a similar rate of iron export between control and prolactin treatment. The increased CD44 by prolactin treatment therefore suggests more iron accumulation. The same upregulation of CD44 by prolactin stimulation was also reproduced with another mouse mammary cancer cell line, Py230 (Supplementary Figure S3C). In Py230 cells, there were more changes of gene expression in iron transport, including downregulation of DMT1 (Supplementary Figure S3B) and upregulation of CD163 (Supplementary Figure S3D) and FPN I (Supplementary Figure S3E). There was also a trend but not significant downregulation of TFRC (Supplementary Figure S3A). The expression of hepcidin remained unaffected (Supplementary Figure S3F) and the ratio of hepcidin/FPN I expression was indifferent between control and prolactin treatment in Py230 cells (Supplementary Figure S3G). In contrast to the results observed in breast cancer cells, prolactin stimulation in the normal mouse mammary epithelial cell line HC11 led to a significant downregulation of CD44 (Figure 2H). To further relate our findings to human breast cancers, we analyzed the gene expression profiles of breast tumors and matched normal breast tissues from 2326 breast cancer patients in the TCGA database. Among these patients, CD44 was frequently mutated, with copy number alterations such as amplification, gain, diploid, shallow, and deep deletion, leading to an average 8.11-fold upregulation of CD44 compared to paired normal tissues (Figure 2I). Moreover, an examination of prolactin expression in tumors revealed a very weak but positive correlation between tumoral prolactin and CD44 expression (Spearman coefficient = 0.05, *p* = 0.038) (Figure 2J).

Taken together, the observed accumulation of iron in mammary cancer cells by prolactin stimulation (seen in Figure 1A) was at least partly mediated through the upregulation of CD44, which transported hyaluronate-Fe. 

### 2.3. CD44 Is Essential for Prolactin-Driven Accumulation of the Labile Iron Pool

**Figure 3:**
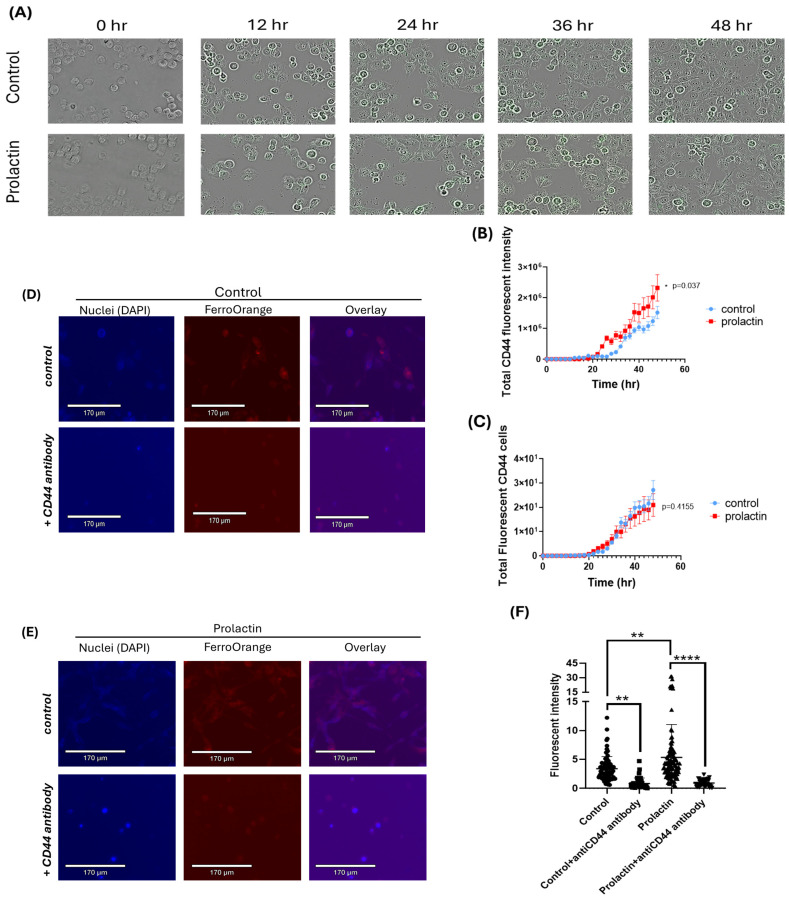
The upregulated CD44 by prolactin stimulation in EO771 cells contributed to iron uptake. (**A**) Representative image of live detection of CD44 expression in cultured EO771 cells over a two-day time course. Green fluorescence indicates the expression of CD44 in EO771 cells. (**B**) Quantification of green fluorescent intensity of CD44 expression per EO771 cell. Data represent mean ± SEM from twelve biological replicates (*n* = 12) (*, *p*< 0.05) (**C**) Percentage of CD44 expressing EO771 cells over two-day time course. Data represent mean ± SEM from twelve biological replicates (*n* = 12). Representative image ((**D**), control (**E**), and Prolactin treatment) and fluorescent quantification of intracellular labile iron pool through blockage of CD44 (**F**). Data represent mean ± SD. (**, *p* < 0.01; ****, *p* < 0.0001). Blue and red fluorescence stain represent the nucleus and labile iron pool, respectively.

CD44, together with other markers, could determine cell stemness. Cancer stem cells have greater requirements for iron uptake and usage than “bulk” cancer cells [18]. Validation of the RNA expression results was confirmed through increased CD44 by prolactin treatment through the detection of live cell expression of CD44 protein. A live cell CD44 antibody with green fluorophore conjugation was added to identify CD44 expression in mammary cancer cells. The intensity of green fluorescence was therefore a reflection of CD44 expression levels on the surface of live, cultured, cancer cells. As seen in Figure 3A,B, an increase in CD44 expression corresponding to the time course by prolactin treatment was observed. In contrast to the enhanced CD44 fluorescence intensity per cell basis, the percentage of cancer cells expressing CD44 was not different over a 2-day period of observation between control and prolactin treatment (Figure 3C), suggesting that prolactin did not induce but enhanced/upregulated CD44 expression in cancer cells. 

To correlate the CD44 expression to iron uptake, we used an antibody to block CD44 and examined the iron uptake in cancer cells following prolactin treatment. As seen in Figure 3D,F, prolactin stimulation led to an increased labile iron pool (upper panel and compared to control), while a low detectable labile iron pool was found in cells when CD44 was blocked by the antibody (lower panel). Similarly, CD44 blockage also decreased labile iron pool in controls (Figure 3D,F). As CD44 was upregulated by prolactin, blocking CD44 in mammary cancer cells would therefore inhibit iron transport, mediated through the CD44. Collectively, these data show that CD44 is involved in prolactin mediated iron uptake in cancer cells.

### 2.4. Increased Iron Export in Macrophages Is Stimulated by Prolactin

The iron concentration in blood is generally low and most iron is recycled [62]. Therefore, cancer cells need to acquire iron through an additional route to satisfy their iron needs. In adults, ~70% of the iron is present as hemoglobin in erythrocytes, ~10% acts as cofactors for various proteins, and ~20% is predominantly carried and stored by macrophages and liver cells [8]. During tumor development, macrophages infiltrate tumors and tumor-associated macrophages (also referred to as M2 macrophages or TAMs) are known to facilitate tumor progression [63]. Thus, we were curious to investigate whether prolactin stimulation impacted the iron uptake/export in macrophages.

**Figure 4:**
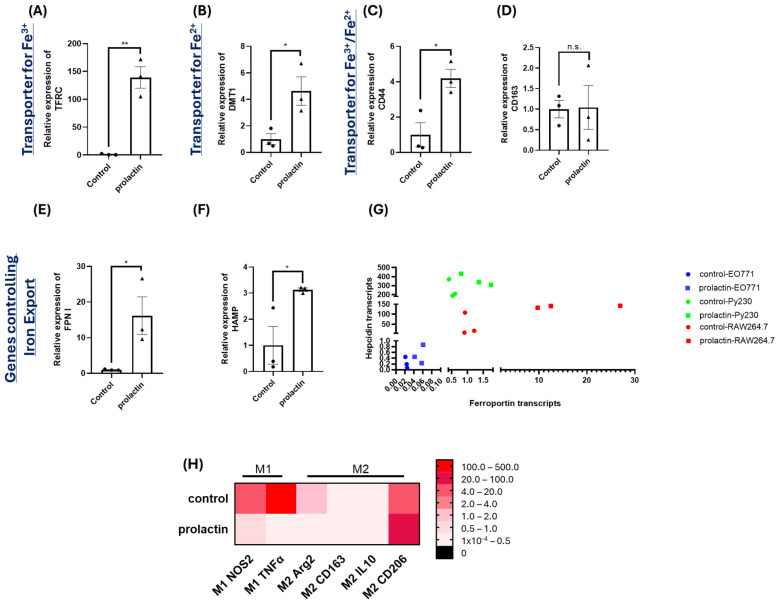
Effects of prolactin treatment on gene expression involved in iron transport in macrophage cell line, RAW264.7. Changes in expression levels in the ferric iron transporter TFRC (**A**), ferrous iron transporter DMT1 (**B**), hyaluronate receptor CD44 (**C**), scavenger receptor CD163 (**D**), iron exporter FPN I (**E**), the negative regulator hepcidin for iron exporter (**F**), and the ratio of hepcidin to FPN I (**G**). The heatmap displays the expression of six genes related to macrophage polarization in response to prolactin treatment, including two M1 markers (NOS2 and TNFα) and four M2 markers (Arg2, CD163, IL10, and CD206) (**H**). Data represent mean ± SEM from three biological replicates (*n* = 3). (*, *p* < 0.05; **, *p* < 0.01; n.s. = not significant) Data represent the relative expressed transcripts of examined iron transport related genes compared to the housekeeping gene, TATA-box binding protein.

Mouse macrophages, RAW264.7, were cultured and exposed to prolactin for 2 days. Real-time q-PCR was performed to analyze gene expression differences in iron transport. As seen in Figure 4, among the same panel of genes examined, there were significant impacts on multiple genes including the upregulation of TFRC, DMT1, CD44, FPN I, and hepcidin, with CD163 being the only unaffected gene by prolactin stimulation. Of note, the upregulation of FPN I by prolactin in macrophages was over 10-fold, significantly higher than that found in EO771 or Py230 cells. These results implied a vigorous iron intake and export by prolactin treatment in macrophages. To better interpret the results, analysis of the raw expression value of each gene was conducted (the relative expressed transcripts to the housekeeping gene, TATA-box binding protein). As seen in Figure 4H, prolactin treatment upregulated FPN I in both cancer cell lines (EO771 and Py230). However, such elevation of FPN I by prolactin was more pronounced in macrophages, suggesting a tendency to export more iron following prolactin stimulation in macrophages. Additionally, the relative expressed transcripts of hepcidin were much higher in the triple negative breast cancer cells (Py230) compared to the luminal B EO771, also suggesting a positive correlation of iron retention and aggressiveness of cancer cells. Macrophages are heterogeneous, comprising various intermediate subtypes with M1 and M2 at the two extremes [64]. The literature indicates that M1 macrophages tend to sequester iron, while M2 macrophages release it [65]. To determine if prolactin-stimulated macrophages affect macrophage plasticity, we examined the expression of genes associated with macrophage polarization, including NOS2 (M1 marker), TNFα (M1 marker), Arg2 (M2 marker), CD163 (M2 marker), IL10 (M2 marker), and CD206 (M2 marker). As shown in Figure 4H, prolactin-stimulated macrophages shifted towards an M2-like subtype, with significant downregulation of NOS2 and TNFα (M1 markers) and upregulation of CD206 (M2 marker).

**Figure 5:**
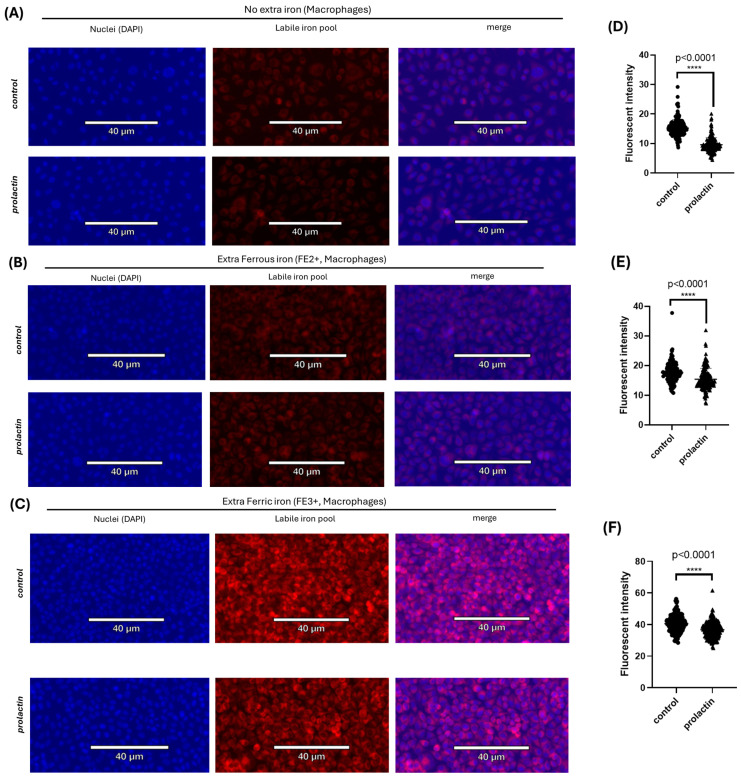
Regulation of intracellular labile iron pool by prolactin in macrophage cells, RAW264.7, under normal culture condition or extra-iron supplementations. (**A**–**C**) Effect of prolactin treatment on the intracellular labile iron pool in RAW264.7 under normal culture condition (**A**), supplemented with extra ferrous iron (reduced state) (**B**), or supplemented with extra ferric iron (oxidized state) (**C**). Blue and red fluorescence stains represent the nucleus and labile iron pool, respectively. (**D**–**F**) Quantification of the intracellular labile iron fluorescent intensity by prolactin treatment in RAW264.7 cells under normal (**D**), extra ferrous (**E**), or extra ferric iron supplementation (**F**). Results are calculated as the average labile iron fluorescence of all individual cells from three independent trials (*n* = 3). Data represent mean ± SD. (****, *p* < 0.0001).

To further confirm these results, we performed labile iron pool staining in macrophages. Prolactin stimulation decreased the labile iron pool in macrophages (fluorescent intensity of 15.23 and 9.59 for control and prolactin treatment, respectively, Figure 5A and Figure 5D). This was also true with the extra supplementation of ferrous or ferric iron (fluorescent intensity of 17.62 and 15.47 for control and prolactin treatment with extra ferrous iron, respectively, and fluorescent intensity of 40.59 and 36.75 for control and prolactin treatment with extra ferric iron, respectively, Figure 5B,E and Figure 5C,F). Extra ferrous or ferric iron supplementation increased labile iron pool in both the control and prolactin groups (fluorescent intensity of 17.62 and 40.59 for extra ferrous and ferric iron supplementation in control, respectively, compared to 15.23 intensity in no extra iron control, and fluorescent intensity of 15.47 and 36.75 for extra ferrous and ferric iron supplementation in prolactin treatment, respectively, compared to 9.59 intensity in no extra iron prolactin group, Figure 5A–F). The numerical number of fluorescent quantifications for RAW264.7 is presented in Table 2.

### 2.5. Macrophages Donate Ferrous Ions Directly from Their Labile Pool to Mammary Cancer Cells

**Figure 6:**
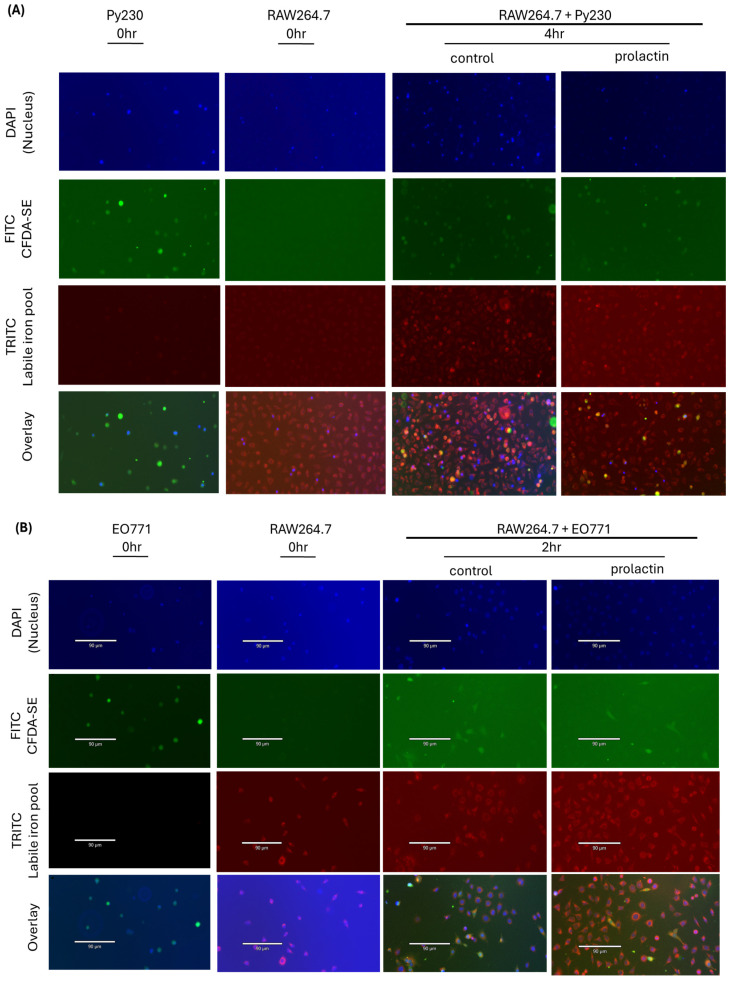
Direct iron transfer from co-cultured macrophages to breast cancer cells. (**A**) Iron transfer occurs in a very short time frame (4 h) between Py230 and RAW264.7 cells. (**B**) Iron transfer occurs in a very short time frame (2 h) between EO771 and RAW264.7 cells. Representative images are selected from three biological replicates. Blue, green, and red fluorescence stains represent nuclei, cytoplasm, and labile iron pools, respectively.

Since prolactin increased iron uptake in the mammary cancer cells and decreased iron contents in macrophages, and as tumor macrophages are known to increase iron export within the tumor microenvironment [66], formulation of the bold hypothesis proposed that macrophages served as direct iron donors by sending their intracellular iron straight into the mammary cancer cells. To answer this question, staining of the intracellular labile iron pool in the macrophages was conducted with FerroOrange (red fluorescence) and nuclei with NucBlue (blue fluorescence). All extraneous stains were then removed and washed to ensure there was no residual dye. Concurrently, mammary cancer cells (Py230) were stained with CFDA-SE (green fluorescence) and nuclei (NucBlue). Next, cells were washed with DPBS after staining to remove residual dye. Addition of the stained mammary cancer cells (green cytoplasm and blue nuclei) into the stained macrophage culture (red labile iron pool and blue nuclei) in serum free medium (to exclude the iron contents present in fetal bovine serum) ensured that the macrophages were the only source of iron. Under co-culture, we observed that all tumor cells became yellowish in color, indicating iron transfer directly from macrophages into cancer cells at 4 h following co-culture (Figure 6A). The iron transfer was less evident at later time points, 24 h and 48 h, due to the tumor phagocytic capacity of RAW264.7 cells (Supplementary Figure S4). The same iron transfer results were also observed using the EO771/macrophage co-culture (Figure 6B). Thus, macrophages acted as direct iron donors.

**Figure 7:**
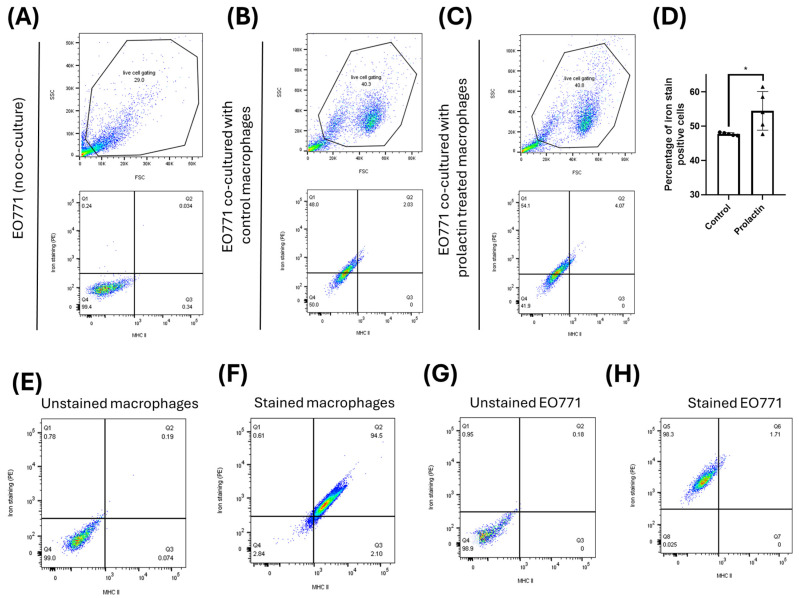
Flow cytometric analysis validates the direct iron transfer from macrophages to breast cancer cells. No fluorescent iron signal was found in EO771 cells when not co-cultured with macrophages (**A**) but iron fluorescence was seen when co-cultured with either control macrophages (**B**) or macrophages pretreated with prolactin (**C**). (**D**) More EO771 cells received fluorescent iron from prolactin pre-treated macrophages. Data represent mean ± SD from five biological replicates (*n* = 5). (*, *p* < 0.05). Staining controls for flow cytometry are shown in (**E**) (unstained macrophages), (**F**) (iron and MHC II stained macrophages), (**G**) (unstained EO771 cells), and (**H**) (iron and MHC II stained EO771 cells).

To further validate the iron transfer results, conduction of a co-culture experiment in which macrophages were first pretreated for 2 days with either prolactin or vehicle control (DPBS), followed by iron staining. These iron-stained macrophages were then co-cultured with non-iron-stained EO771 cells for 2 h, after which the macrophages were removed to prevent further phagocytosis of tumor cells. The EO771 cells were subsequently collected for flow cytometry analysis to detect any fluorescent iron transferred from macrophages. To ensure that the iron fluorescence was not originating from residual macrophages that were not entirely washed away, MHC II staining was used, as MHC II is predominantly expressed by antigen-presenting phagocytes. As shown in Figure 7A, EO771 cells not co-cultured with macrophages exhibited neither fluorescent iron nor MHC II expression, the latter being a specific marker for phagocytes. When EO771 cells were co-cultured with DPBS-treated macrophages, 47.72% of the EO771 cells became fluorescent iron positive while remaining MHC II negative (Figure 7B). Co-culturing with prolactin-pretreated macrophages increased this proportion to 54.47%, with the EO771 cells still remaining MHC II negative (Figure 7C). This result aligns with previous findings that prolactin enhances iron release from macrophages (Figure 4H and Figure 5), leading to more EO771 cells acquiring iron from prolactin-pretreated macrophages (Figure 7D). MHC II expression, specific to antigen-presenting phagocytes, was only observed in macrophages, not in EO771 cells (Figure 7F,H).

## 3. Discussion

A tumor is a heterogeneous tissue consisting of different stromal, epithelial, endothelial cells, and tumor cells [67,68]. Additionally, small molecules, endocrine factors, and cytokines further contribute to the complexity of the tumor [69,70]. The ferrophilic feature of cancer cells make iron transport and metabolism ideal targets for potential cancer therapy. However, how the iron transport and metabolism are precisely regulated in the confined tumor microenvironment is not well understood. Prolactin is a peptide hormone produced by the anterior pituitary gland [31], but evidence also shows non-lactotroph prolactin can be produced by cancer cells [36,71,72] and such prolactin within the tumor microenvironment is positively correlated to tumor progression [37,39,40,41,53]. Interestingly, prolactin elevation is noted in patients with iron deficiency [47,48]. However, the exact role of prolactin in iron regulation is completely unknown. To dissect the impact of prolactin on cellular iron regulation, examination of the iron transport dynamics between mammary cancer cells and macrophages stimulated by prolactin is needed. Results show distinct roles of prolactin in mammary cancer cells and macrophages. In mammary cancer cells, prolactin causes elevation of intracellular ferrous iron pools (also known as labile iron pools) through the upregulation of the surface receptor CD44. In contrast, prolactin stimulation has the opposite impact on macrophages, i.e., to promote the iron release from macrophages. With a specialized co-culture, macrophages can transfer their intracellular iron to mammary cancer cells. Thus, this current work provides new insight into how prolactin impacts cancer progression through its novel role in iron redistribution within the tumor microenvironment.

While prolactin is well recognized for its roles in lactation and tumor progression, its involvement in iron homeostasis has remained elusive. This study marks the first exploration of prolactin’s novel role in mediating iron distribution between two distinct cell types: cancer cells and macrophages. Several studies have provided some insights into the potential links between prolactin and iron metabolism in humans. Notably, iron deficiency has been associated with elevated serum prolactin levels and subsequent dopaminergic neuron defects [49,50]. Moreover, iron deficiency enhances prolactin binding to liver cells, although the regulatory role of prolactin in hepatic iron regulation remains uncertain—the liver being the primary organ governing iron homeostasis [73,74]. Additionally, studies by Wang et al. have suggested that patients with hyperprolactinemia exhibit lower levels of hepcidin, implying a possible role for prolactin in iron release [48]. Furthermore, Kupffer cells, specialized macrophages in the liver, play a crucial role in iron homeostasis depending on their activation state. Classical activated macrophages (M1) tend to sequester iron, while alternatively activated macrophages (M2) appear to release iron [65]. A correlation between M2 polarization and hyperprolactinemia has been reported [75]. In this current study, direct evidence linking these previous findings together is provided. Direct stimulation of macrophages by prolactin induces iron export, likely mediated by the pronounced upregulation of ferroportin in macrophages. Additionally, prolactin-treated macrophages displayed a M2-like subtype, characterized by significant upregulation of CD206. Furthermore, cancer cells serve as major recipients for the released iron, facilitated by the upregulation of CD44—a consistent finding across both tested breast cancer cell lines and human breast cancer patients from the TCGA database. 

Previous studies have indicated co-culture of macrophages and cancer cells increases iron content in cancer cells. This is regulated by the adipocytokine lipocalin 2 (LCN2), which released by macrophages, and its neutralization reverses iron elevation in cancer cells [76]. However, it is not known whether cancer cells acquire iron directly from macrophages or from culture medium that contains ~6 µM iron after being stimulated by LCN2. To address this issue, this study employed a co-culture system in which macrophages with pre-stained labile iron pools were cultured alongside cancer cells that were not stained for iron. Results showed translocation of the pre-stained iron, previously inside the macrophages, to the cancer cells. Hence, we provide first-hand evidence of direct transfer of ferrous iron from labile iron pools inside the macrophages to mammary cancer cells. 

Iron exists in two different states, either the reduced form (ferrous iron (Fe^2+^)) or the oxidized form (ferric iron (Fe^3+^)). While current literature does not specify which state cancer cells are “addicted” to, ferric iron is insoluble and is often coupled to protein chaperones (e.g., transferrin) for its transport [77]. As mentioned earlier, TFRC is the major receptor for Fe^3+^, DMT1 for Fe^2+^, and CD163 and CD44 are receptors for both states. Presented in the work here, EO771 cells appear to prefer Fe^3+^ as extra Fe^3+^ increases their intracellular labile iron pool. While ferric iron supplementation significantly increases the labile iron pool in EO771 cells, adding extra iron, whether ferric or ferrous, does not affect tumor cell proliferation. Therefore, the addition of different iron states and the size of the labile iron pool may not be the sole determinants of tumor cell activity, at least in terms of cell proliferation. With the data collected from Py230 cells, there was no such preference for Fe^2+^ or Fe^3+^, since the extra supplementation of both iron forms does not affect labile iron pools significantly. Consequently, the preference for each iron state might be largely dependent upon the cell and the state of iron supplementation is not the only factor affecting the size of labile iron pool. However, prolactin stimulation in both mammary cancer cell lines significantly increases labile iron pools via the upregulation of CD44. The labile iron pool is a cellular pool of chelatable iron complexes (mostly in ferrous form) [78] and CD44 mediates the endocytosis of iron (both ferrous and ferric iron) bound to hyaluronan [27,79]. Thus, the increase in the labile iron pool by prolactin stimulation might be due to (i) more direct uptake of ferrous iron bound hyaluronan or (ii) increased ferric iron reduction by the ferrireductase STEAP into ferrous iron after uptake of ferric iron bound hyaluronan. Moreover, iron can form complexes with various compounds such as fumarate, sulfate, gluconate, lactate, succinate, citrate, pyrophosphate, hyaluronate, or carbohydrate coatings like sucrose. It remains to be determined whether prolactin stimulation leads to the generation of metabolites or the release of pyrophosphate or other compounds that could complex with iron, and how the degree of ionization of these complexes in the co-culture might influence the CD44 pathway. Future work focusing on the iron re/dox and iron complexes may provide more knowledge in the prolactin driven iron transport/metabolism. 

CD44 is a cell surface receptor involved in many activities such as cell-cell interaction, cell-extracellular matrix interaction, and cellular mobility [80]. It is reported as a receptor that impacts iron homeostasis through hyaluronate-dependent iron endocytosis pathway. CD44, together with other biomarkers, serves as a marker for stemness evaluation [81,82]. CD44 is also expressed by many different cell types, including epithelial cells and immune cells [83,84,85]. Thus, CD44 expression is important to multiple physiological processes. CD44 knockout animals have shown impacts on lymphocyte circulation [86] and CD44 knockout tumor cells have significantly decreased proliferation, survival in vitro, and a slowed tumor development when implanted into mice [87,88]. In the current study, using CD44 neutralizing antibodies significantly decreases iron content in breast tumor cells but this could also be subject to indirect impact on cellular proliferation and survival. CD44 exists in multiple variants due to alternative splicing and it is recognized that different spliced isoforms may contribute differently to tumor progression [88,89]. As hyaluronan is the major ligand for the CD44 receptor, it is unclear which spliced isoform of CD44 would be responsible for the iron-bound hyaluronan, thus regulating iron transport. Further investigation on the binding of different iron state-coupled hyaluronan to different CD44 spliced variants through knockout/knockdown of specific spliced variant(s) may uncover novel insights for therapeutic interventions. 

While this study shows direct iron transfer from labile iron pool inside the macrophages into mammary cancer cells, there are limitations to the study. Ongoing studies include exploring a triple culture of macrophage, mammary cancer, and normal mammary cells to investigate the dynamics of iron uptake in a more heterogeneous population of cells. Future studies should include prolactin overexpressing cells as controls and the detailed mechanism(s) underlying this transfer require further investigation. For instance, will the macrophages release ferrous iron into the environment, that is then taken by cancer cells directly via DMT1? Alternatively, will macrophages release ferrous iron into the environment to be immediately coupled with hyaluronan prior to uptake by the cancer cells through CD44? Finally, this study focused on the uptake of iron to the mammary cancer cells. Addressing the fate of the macrophages once the iron stores are released needs further investigation. As macrophages are one type of tumor infiltrating immune cells and M2 macrophages are known to facilitate tumor progression and metastasis, understanding how iron transport is managed between macrophages and cancer cells may further assist the therapeutic design to interrupt the tumor progression.

In the iron transfer experiment, gradually decreased “intact” cancer cells present at 24 and 48 h are noted. This is due to the phagocytic capacity of RAW264.7 [90,91]. However, as macrophages are pre-treated with prolactin for 2 days before the co-culture with tumor cells for iron transfer, some macrophages shift into M2-like phenotype, thereby exhibiting iron transfer capacity. Therefore, a fully polarized M1 macrophage population with tumor phagocytic activity might inhibit iron transfer. However, as the tumor progresses, particularly in a real tumor microenvironment, iron transfer would likely become more pronounced as macrophages fully transition to M2 or M2-like subtypes. In the iron transfer experiment, macrophages are cultured and treated with prolactin in complete medium, ensuring that gene expression related to iron transfer reflects the prolactin treatment but not the presence or absence of serum. Since serum contains a large amount of iron, approximately 25 times more than intracellular labile iron, its presence could interfere by acting as a significant iron source for tumor cells, competing with the iron transferred from macrophages. To address this, macrophages are switched to serum-free conditions before being co-cultured with tumor cells. A short period in serum-free conditions is unlikely to significantly impact gene expression or cellular activities, and we still observe iron transfer between macrophages and tumor cells. However, after 24 to 48 h in serum-free conditions, cellular activities and gene expression may be affected. This, combined with the macrophages’ phagocytic capacity, may explain why we do not see a dramatic difference in iron transfer between control and prolactin-treated groups at 24 and 48 h.

While iron has been shown to positively regulate tumor growth, the iron dependent growth analyses presented in this study are not significant. There are two likely reasons for these results. First, aqueous ferric iron is insoluble. Although some ferric iron added into culture might be readily coupled with transferrin, most ferric iron forms insoluble ferric oxide precipitates in the presence of oxygen, rendering it less biologically accessible [92]. Thus, there is less significant impact on the dose related growth. Second, free ferrous iron is known to catalyze the formation of toxic oxygen radicals via Fenton chemistry [93,94], thus producing cytotoxicity. Therefore, the observed cellular growth may likely be a combinational result of cell growth and cell toxicity, leading to less significant growth curves. 

## 4. Materials and Methods

### 4.1. Cell Culture and Treatment

Mouse mammary cancer EO771 cells (CRL-3461TM, ATCC, Manassas, VA, USA), Py230 (CRL-3279™, ATCC, Manassas, VA, USA) (referred to collectively as mammary cancer cells), and the mouse macrophage cell line RAW264.7 (TIB-71, ATCC, Manassas, VA, USA) were cultured in RPMI 1640 (10-040-CV, Corning, Corning, NY, USA) with 10% FBS (10082-147, ThermoFisher, Waltham, MA, USA) and 1% penicillin plus streptomycin supplement (SV30010, Citiva, Marlborough, MA, USA) at 37 °C in 5% CO_2_ under humid conditions. Py230 is a known triple negative mammary cancer (TNBC) cell line with no estrogen receptor (ER) or progesterone receptor (PR) and very low Her2 expression. EO771 is controversial in classification. Most studies define EO771 as luminal type B mammary cancers [95,96] while other studies indicate EO771 as TNBC since the growth of EO771 is independent of estradiol stimulation and the ER expression is limited to cytoplasm but not nucleus [97,98]. The physiological concentration of iron ranges from approximately 60–180 µg/dL (0.6–1.8 µg/mL) and many cancer patients exhibit iron deficiency anemia [99]. In fetal bovine serum (FBS), the iron concentration is about 2.4 µg/mL. In this study, cells were cultured in medium containing 10% FBS, resulting in a final iron concentration of ~0.24 µg/mL to mimic iron deficient condition in cancer patients. To test cellular responses to additional ferrous or ferric iron, cells were treated with 10 µg/mL or 100 µg/mL to examine the impact on cellular growth (Figure S1E,F) and with 100 µg/mL to investigate cellular iron uptake (Figure 1B,C and Figure 5B,C). The serum prolactin level is approximately 25 ng/mL in females and ~20 ng/mL in males. Serum prolactin is positively correlated with breast tumor progression, and the average serum prolactin concentration in breast cancer patients is ~102.68 ± 7.03 ng/mL [100]. Therefore, 100 ng/mL prolactin was used to mimic conditions in cancer patients. Cell growth in response to extra iron (either ferrous or ferric at different concentrations) was measured by direct cell count.

### 4.2. Gene Expression Analyses in Breast Cancer Cell Lines, Macrophages, Normal Mammary Cell Line and Human Breast Cancer Patients 

To explore the impact of prolactin on iron transport in the macrophage cells and mammary cancer cells, expression of appropriate genes was analyzed by real-time PCR. Briefly, 5 × 10^5^ cells (macrophages or mammary cancer cells) were seeded in 6 well plates and treated with 2 µL of DPBS (as control) or 2 µL of 100 µg/mL prolactin (SRP9000, Sigma-Aldrich, St. Louis, MO, USA), the final working concentration of prolactin is 100 ng/mL) for 2 days. Cells were harvested, RNA was extracted using Trizol (15596026, Invitrogen, Waltham, MA, USA) following manufacturer’s instruction, and cDNA synthesis was conducted using a RevertAid RT Reverse Transcription Kit (K1691, ThermoFisher, Waltham, MA, USA). Real-time PCR was conducted on a Chai open qPCR system (Chai, Santa Clara, CA, USA). Primers used for examining gene expression involved in iron transport and macrophage polarization are listed in Table 3. Gene expression analyses were normalized to the housekeeping control, mouse TATA-box binding protein (TBP). The gene expression profile of prolactin-stimulated normal mammary epithelial cell line, HC11, was retrieved from the GEO database (GSE107419) [101]. Genes involved in iron transport, which were also analyzed in breast cancer cell lines, were normalized to TBP expression, and their relative expression was compared to assess the impact of prolactin stimulation. To relate our findings to human cancers, we analyzed data from 2326 breast cancer patients diagnosed with either invasive lobular or ductal carcinoma using the TCGA database. This same patient cohort was also studied in other literature [102,103,104]. For these patients, both cancerous and matched normal tissues were collected, and gene expression profiles in cancer tissues were normalized against their matched normal tissues. The analyses were conducted using cBioPortal and included mutation types, copy number variations, and expression intensity in cancer tissues after normalization. Additionally, the correlation between tumoral expression of CD44 and prolactin was assessed using Spearman rank correlation.

### 4.3. Labile Pool Iron Staining (FerroOrange Stain)

To visualize and determine the presence and uptake of iron in cells, FerroOrange staining (SCT210, Sigma-Aldrich, St. Louis, MO, USA) was performed. Briefly, 1 × 10^5^ of cells were seeded in a 60 mm petri dish. On the second day, cells were washed twice with DPBS to remove any free iron from the culture medium. Cells were then stained with 0.3 µM of FerroOrange staining reagent and incubated at 37 °C for 30 min. After incubation, cells were washed with DPBS again to remove extraneous FerroOrange dye and fresh serum-free RPMI1640 (containing no iron) was added to cells for downstream microscopic observation. FerroOrange dye stains the ferrous (Fe^2+^) ions that are present within the labile pool in cells and fluoresces red with 530 nm excitation and 572 nm emission. Fluorescence observations were performed using an Echo Revolve Fluorescent Microscope (Echo, San Diego, CA, USA). Iron staining fluorescence was quantified using ImageJ (version 1.54i, developed by the National Institutes of Health and the Laboratory for Optical and Computational Instrumentation). Briefly, a square region of the same size was identified in the center of each image. The cells of interest within this region were selected, and their fluorescence intensity was measured. Background values were determined from non-fluorescent regions adjacent to the selected cells. After correcting for background, the values from all trials were combined. The corrected total cell fluorescence (CTCF) was calculated using the following formula and CTCF values for each group were then plotted using GraphPad Prism software (Version 8.01).

CTCF = Integrated Density − (Area of selected cell × Mean fluorescence of background readings).

### 4.4. Determination of Iron Transfer between Macrophages and Mammary Cancer Cells 

To investigate iron transfer between macrophages and mouse mammary cancer cells, co-culture of macrophages and mammary cancer cells were conducted. Briefly, 1 × 10^5^ of RAW264.7 cells were seeded in two 60 mm petri dishes 24 h before the experiment. The next day, macrophages were stained with FerroOrange dye as described above. After staining, one dish of stained macrophages was treated with DPBS as control while the other dish of stained macrophages was treated with 100 ng/mL prolactin in complete medium with serum for 2 days. Following this 2-day treatment, the medium was removed, the macrophages were washed, and then re-cultured in serum-free medium. This ensures that the macrophages will be the sole source of iron, eliminating the potential interference of serum iron in the study of iron transfer.

Mouse mammary cancer cell suspensions (EO771 and Py230) were stained with CFDA-SE (V12883, ThermoFisher, Waltham, MA, USA) and NucBlue Live Cell stain (R37605, ThermoFisher, Waltham, MA, USA ) for 20 min in 37 °C incubator. After staining, cells were centrifuged and washed with DPBS twice to remove any residual CFDA-SE dyes. The final cancer cell pellets were resuspended in serum-free RMPI 1640 medium and 1 × 10^5^ of stained cancer cells (CFDA-SE stains the cytoplasm green and NucBlue stains the nucleus blue) were added to the serum-free macrophage culture described above. Thus, the macrophages contained red fluorescent iron and blue nuclei while the mammary cancer cells were fluorescent green with blue nuclei. The transfer of red fluorescent ferrous iron between macrophages and mouse mammary cancer cells was monitored at 2 h, 24 h, and 48 h after the commencement of co-culture. 

### 4.5. CD44 Expression Analysis by Cellcyte X

Live cell CD44 expression was performed using the CD44 Alexa Fluor™ 488 Conjugate Kit (A25528, ThermoFisher, Waltham, MA, USA). Briefly, 5000 cancer cells were seeded in a 96 well plate with 2 µL of DPBS (as control) or 2 µL of 100 µg/mL prolactin (the final working concentration of prolactin is 100 ng/mL). A 1:50 volume of the dye-conjugated antibody directly to the cell culture medium. The plate was then placed on the Cellcyte X (CYTENA, Boston, MA, USA) to record the CD44 expression over time every 2 h till the end of the 3-day experimental period. The Cellcyte X is a high-throughput live-cell imaging system that enables real-time fluorescent imaging of live cells directly within the incubator. It has built-in software that converts real-time fluorescence data into quantitative values and includes protocols for various analyses such as cell confluency, cell counting, apoptosis and cytotoxicity assays, transfection efficiency, spheroid growth analysis, and immune cell cluster formations. This system was used to measure and quantify the effect of prolactin on tumor cell proliferation and CD44 expression. For each trial, a fluorescent value was obtained at each time point, and the data presented are the results from 12 repeated trials (*n* = 12).

### 4.6. CD44 Neutralization Assay

To evaluate the importance of CD44 expression in the mediation of iron transport in mammary cancer cells, CD44 neutralization assay was conducted. Mouse mammary cancer cells were seeded in 35 mm petri dishes and treated with DPBS (control), DPBS + 15 µg CD44 monoclonal antibody (10759-114, VWR, Radnor, PA, USA), 100 ng/mL prolactin, and 100 ng/mL prolactin + 15 µg CD44 monoclonal antibody for 2 days. Cells were then stained with FerroOrange and NucBlue live cell dyes as described above. 

### 4.7. Flow Cytometry 

To determine the iron transfer between macrophages and tumor cells, macrophages (RAW264.7) were first cultured with or without 100 ng/mL prolactin treatment in complete medium with serum for 2 days. The prolactin treated or untreated macrophages were stained with FerroOrange to label their intracellular labile iron. Macrophages were then collected by accutase and washed with fresh medium to remove any residual stains. The collected suspended macrophages were then added to unstained EO771 cell culture for 2 h in serum free medium. At 2 h, the suspended macrophages were removed and the remaining adherent EO771 cells were analyzed by flow cytometer (BSN-ZS-AS7S, Conduct Science, Skokie, IL, USA) to determine the iron transfer from macrophages. To further validate the cell identity, PE-Cy7-rat against mouse MHC II antibody (25-5321-82, FisherScientific, Waltham, MA, USA) was used. MHC II is expressed in phagocytic immune cells and therefore serves as a macrophage marker. Unstained macrophages and EO771 cells and FerroOrange, PE-Cy7-MHC II labelled macrophages, and EO771 cells were used as internal controls.

### 4.8. Statistical Analysis

All figures are presented as mean ± SEM. Results were considered significant when *p* ≤ 0.05. All graphs were generated using GraphPad Prism software (version 8.01). Briefly, statistical analysis was generally performed by paired *t*-test for comparison between two groups. If more than two groups and data were compared, significance was determined by one way ANOVA with post-hoc Tukey HSD tests. Two-tailed analysis was used for all data analyses.

## 5. Conclusions

The tumor microenvironment is a complex and continuously evolving entity. The elegant regulation between cells and molecules determines the fate of tumor progression. In this study, we present a multidisciplinary regulation of iron transport by an endocrine hormone, immune cells, and cancer cells. Stimulation by prolactin enhances iron accumulation in cancer cells but induces iron release from macrophages. CD44 plays an important role in the prolactin-driven accumulation of labile iron pools inside cancer cells. In addition, macrophages serve as direct iron donors, contributing their iron to cancer cells. As macrophages are key cells maintaining the iron homeostasis via serving as scavenger cells that consume hemoglobin during red blood cell clearance, and as they are migratory cells that often infiltrate tumors, the direct iron transfer between macrophages and cancer cells may provide an efficient way to secure an iron source for cancer cells during disease progression. 

## Figures and Tables

**Table 1:** Fluorescent Intensity of Labile Iron Pool Staining in prolactin treated breast cancer cells.

(No Extra Iron)	EO771	Py230
Control	7.58 ± 6.158	15.96 ± 2.665
Prolactin	15.67 ± 7.43	19.48 ± 6.119

**Table 2:** Fluorescent Intensity of Labile Iron Pool Staining in RAW264.7.

	No Extra Iron	Extra Ferrous Iron	Extra Ferric Iron
Control	15.23 ± 2.844	17.62 ± 3.289	40.59 ± 5.491
Prolactin	9.59 ± 2.435	15.47 ± 3.573	36.75 ± 4.889

**Table 3:** Primers used in qPCR gene expression analyses.

TBP	Forward 5′-GGGTATCTGCTGGCGGTTT-3′ Reverse 5′-TGAAATAGTGATGCTGGGCACT-3′
TFRC	Forward 5′-TTCGCAGGCCAGTGCTAGG-3′ Reverse 5′-GAGTACCCCGACAGCCGTTC-3′
DMT1	Forward 5′-AAGATGCCAGACGATGGCG-3′ Reverse 5′-GAGTTGCTGTAGGCAGGGTT-3′
CD163	Forward 5′-CCTCTGCTGTCACTAACGCT-3′ Reverse 5′-CAGTTGTTTTCACCACCCGC-3′
CD44	Forward 5′-GAGCACCCCAGAAAGCTACA-3′ Reverse 5′-TGAGTGCACAGTTGAGGCAA-3′
FPN I	Forward 5′-AGACAAAAAGAAGACCCCGTGA-3′ Reverse 5′-CACAACAGCCTTATGCCGAA-3′
HAMP	Forward 5′-AGAGAGACACCAACTTCCCCA-3′ Reverse 5′-GCAACAGATACCACACTGGGA-3′
NOS2	Forward 5′-CAGGGAGAAAGCGCAAAACAT-3′ Reverse 5′-CATTCTGTGCTGTCCCAGTGA-3′
TNFα	Forward 5′-TGGCCTCCCTCTCATCAGTTC-3′ Reverse 5′-CCATCTCATCCCATGCCTAACT-3′
CD206	Forward 5′-ACGAGCAGGTGCAGTTTACA-3′ Reverse 5′-ACATCCCATAAGCCACCTGC-3′
Arg2	Forward 5′-GTAATCCCCTCCCTGCCAATC-3′ Reverse 5′-TCAGGACGCAAGGAATTTGC-3′
IL10	Forward 5′-CCTGGGTGAGAAGCTGAAGAC-3′ Reverse 5′-TGTAGACACCTTGGTCTTGGA-3′

## Data Availability

The dataset supporting the conclusions of this article is available from the corresponding author.

## Supplementary Materials

The following supporting information can be downloaded at: https://www.mdpi.com/article/10.3390/ijms25168941/s1.
