# Supplementary material for: Prolactin Drives Iron Release from Macrophages and Uptake in Mammary Cancer Cells through CD44
Source: Int J Mol Sci. 2024 Aug 16;25(16):8941. doi: 10.3390/ijms25168941 (PMC11354873; doi:10.3390/ijms25168941)
Supplement: Supplementary file 1 [file ijms-25-08941-s001.zip › ijms-3153608-supplementary.pdf]

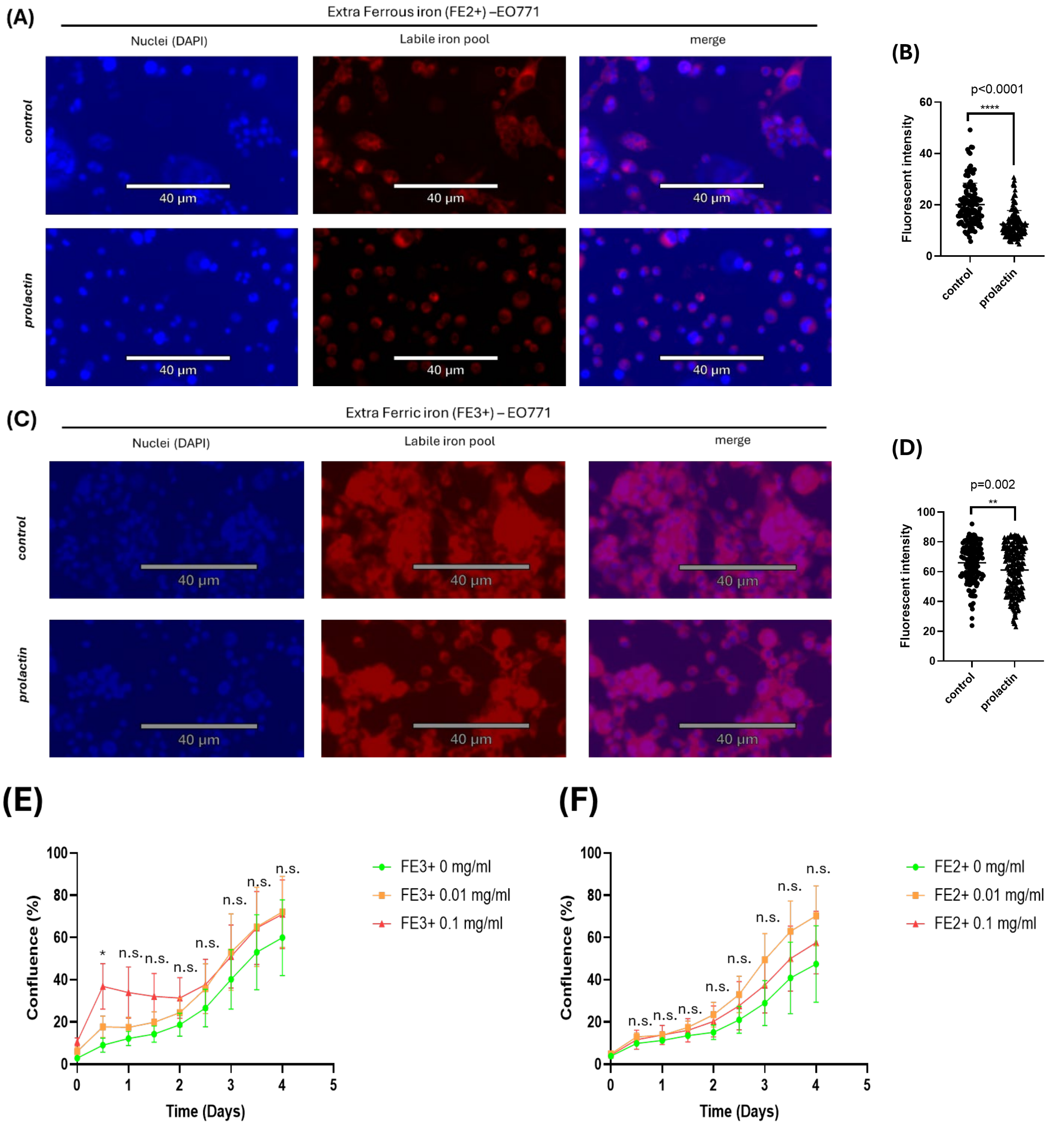

Supplementary Figure S1. Regulation of intracellular labile iron pool by prolactin in mouse breast cancer EO771 cells with extra-ferrous (A) or extra-ferric iron (C) supplementations. Blue and red fluorescence stains nucleus and labile iron pool respectively. Quantification of the intracellular labile iron fluorescent intensity by prolactin treatment in EO771 cells with extra ferrous (B), or extra ferric iron supplementation (D). Proliferative effects on EO771 cells with varying concentrations of extra ferric iron (E) and ferrous iron (F) supplementation.. Data represent mean ± SD. (*, $p<0.05$; **, $p<0.01$; ****, $p<0.0001$, n.s., not significant) .

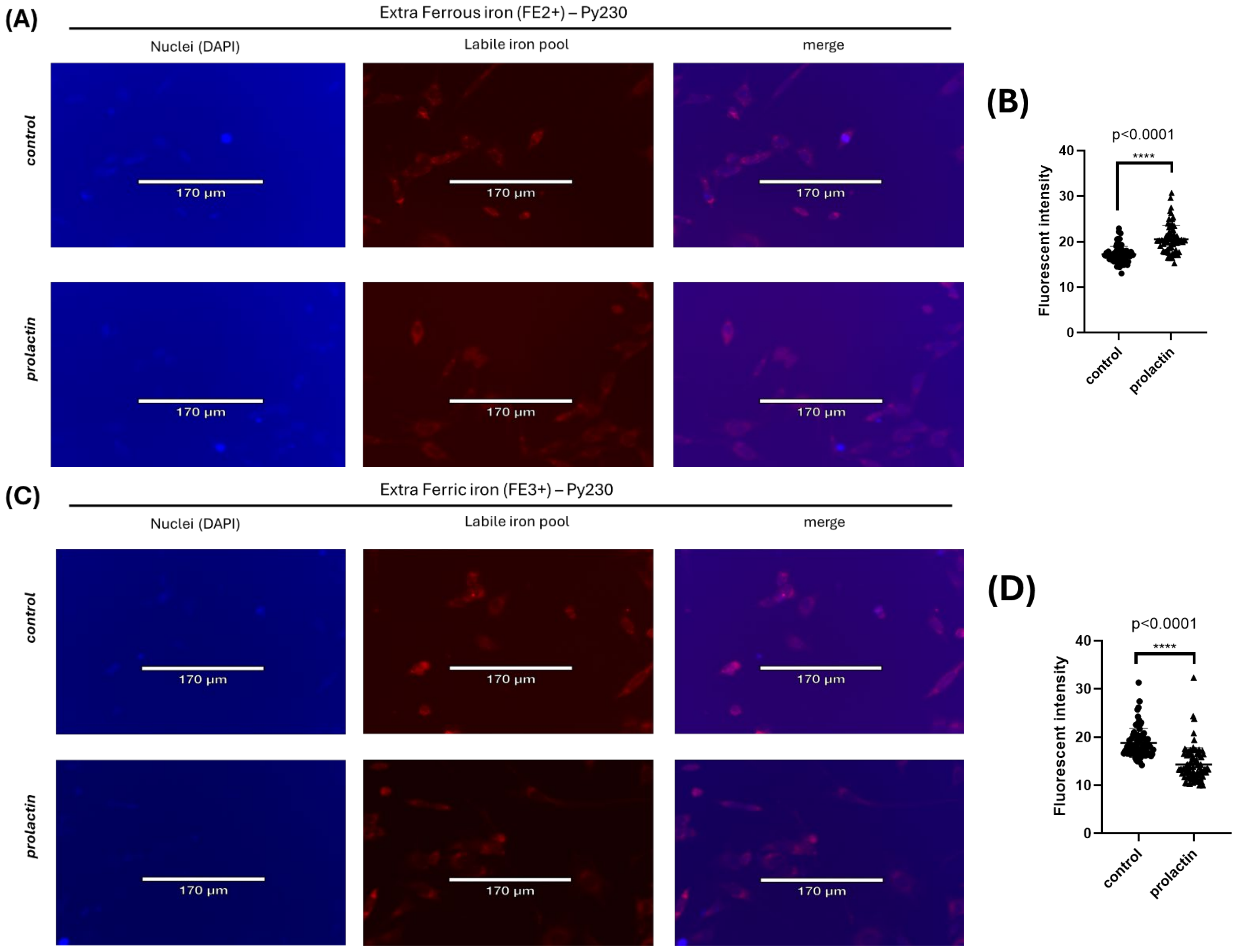

Supplementary Figure S2. Regulation of intracellular labile iron pool by prolactin in mouse breast cancer Py230 cells with extra-ferrous (A) or extra-ferric iron (C) supplementations. Blue and red fluorescence stains nucleus and labile iron pool respectively. Quantification of the intracellular labile iron fluorescent intensity by prolactin treatment in Py230 cells with extra ferrous (B), or extra ferric iron supplementation (D). Data represent mean ± SD. (****, $p<0.0001$)

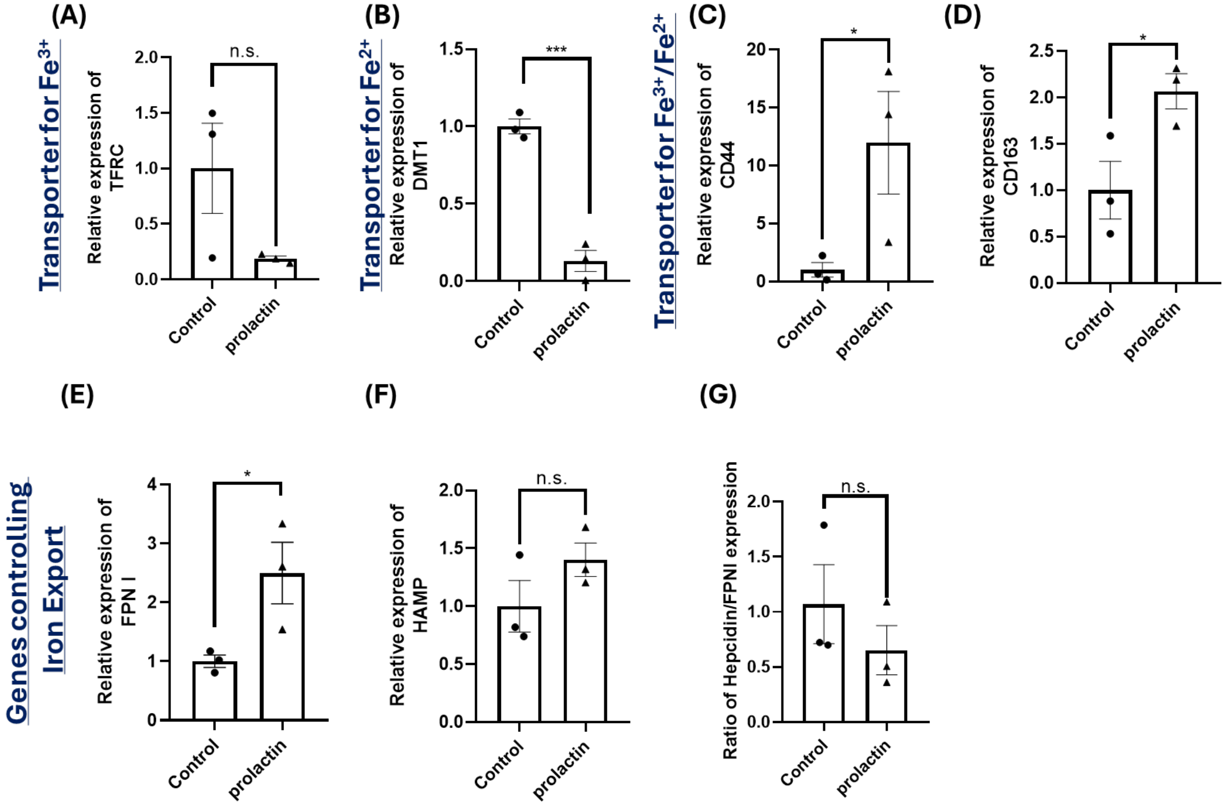

Supplementary Figure S3. Effects of prolactin treatment on gene expression involved in iron transport in Py230 cells. Changes in expression levels in the ferric iron transporter TFRC (A), ferrous iron transporter DMT1 (B), hyaluronate receptor CD44 (C), scavenger receptor CD163 (D), iron exporter FPN I (E), the negative regulator hepcidin for iron exporter (F), and the ratio of hepcidin to FPN I (G). Data represent mean ± SEM from three biological replicates (n=3). (*, $p < 0.05$; ***,
$p < 0.001$; n.s., not significant)

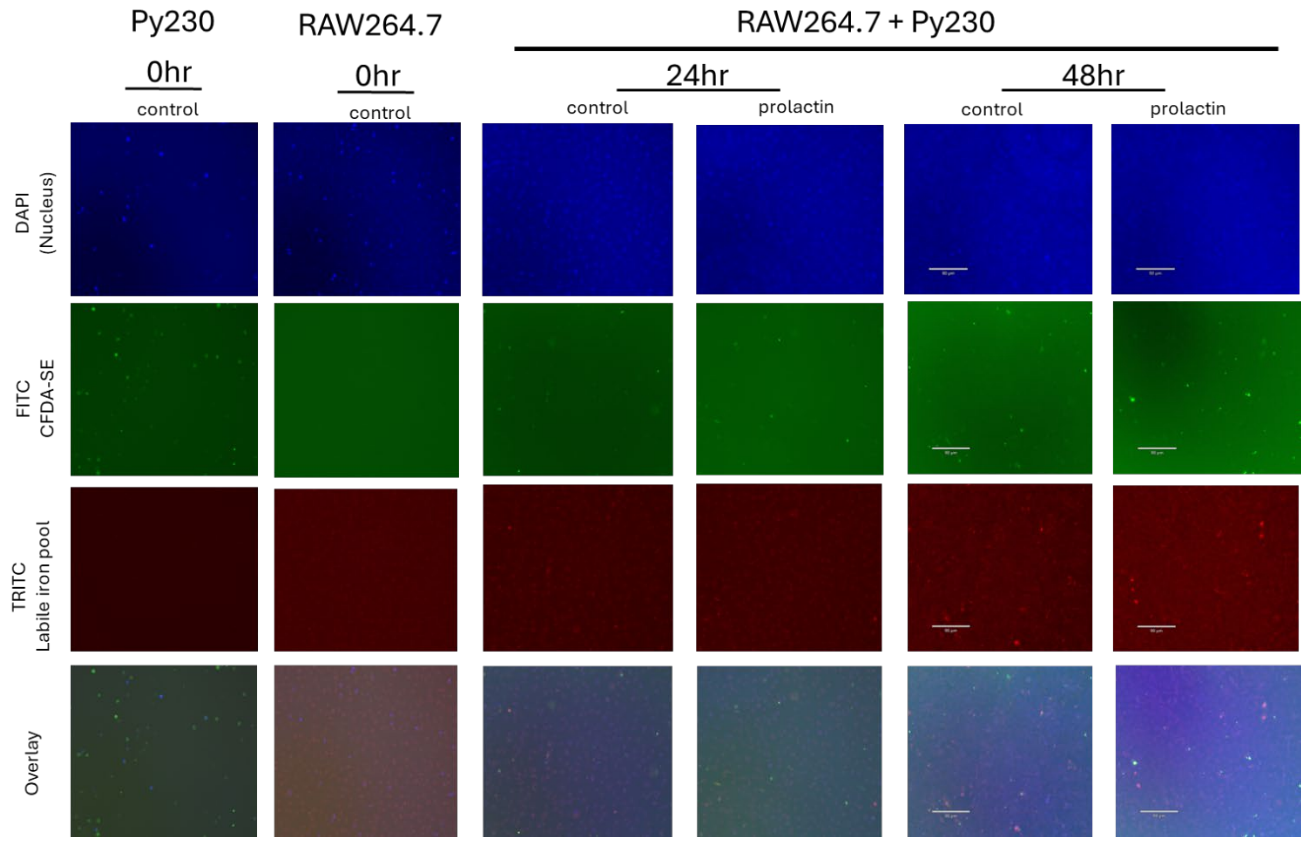

Supplementary Figure 4. Iron transfer between long term co-cultured macrophages to breast cancer cells. Iron transfer also occurs between long term (24 and 48 hours) co-cultured between Py230 and RAW264.7 cells. Representative images are selected from three biological replicates. Blue, green, and red fluorescence stains nuclei, cytoplasm, and labile iron pools respectively.
